# Comparative efficacy of six monthly doses of Simparica Trio^®^ (sarolaner, moxidectin, and pyrantel chewable tablets) versus NexGard^®^ Plus (afoxolaner, moxidectin, and pyrantel chewable tablets) against a macrocyclic lactone-resistant *Dirofilaria immitis* isolate in dogs

**DOI:** 10.1186/s13071-026-07402-4

**Published:** 2026-05-05

**Authors:** Jessica Rodriguez, Shelby Jones, Sean Mahabir, Utami DiCosty, Abdelmoneim Mansour, Crystal Fricks, John W. McCall, Thomas Geurden

**Affiliations:** 1 Zoetis, Veterinary Medicine Research and Development, Kalamazoo, MI USA; 2 TRS Labs Inc, Athens, GA USA; 3 Zoetis, Veterinary Medicine Research and Development, Zaventem, Brussels, Belgium

**Keywords:** Canine, *Dirofilaria immitis*, Macrocyclic lactone, Moxidectin, NexGard^®^ Plus, Resistance, Simparica Trio^®^, ZoeLA

## Abstract

**Background:**

The emergence of macrocyclic lactone (ML)-resistant *Dirofilaria immitis* highlights the need for informed and considered use of currently available heartworm preventives. Moxidectin provides robust protection against multiple ML-resistant *D. immitis* isolates, with efficacy dependent upon both the dosage and the number of monthly treatments administered. In this study the efficacy of six monthly treatments of Simparica Trio^®^ (24 µg/kg moxidectin, 1.2 mg/kg sarolaner, and 5 mg/kg pyrantel pamoate) was compared with NexGard^®^ Plus (12 µg/kg moxidectin, 2.5 mg/kg afoxolaner, and 5 mg/kg pyrantel pamoate), both administered at the recommended label dose.

**Methods:**

A total of 24 dogs were randomly allocated to negative control, NexGard Plus, or Simparica Trio groups. Each dog was inoculated with 50 ML-resistant *D. immitis* L3 larvae (ZoeLA isolate) on day −30. All dogs were dosed orally on days 0, 30, 60, 90, 120, and 150. Upon study completion (day 237), all adult worms were recovered and counted.

**Results:**

Mean moxidectin dosages administered to each group were 17.6 ± 1.6 µg/kg for NexGard Plus and 37.9 ± 8.4 µg/kg for Simparica Trio. Geometric mean (GM) adult worm counts in both NexGard Plus (0.7) and Simparica Trio (0.2) groups were significantly lower than negative control (*P* < 0.0001), with NexGard Plus and Simparica Trio providing 98.1% and 99.5% efficacy, respectively. However, *D. immitis* adult worms were recovered in 6/8 (75%) NexGard Plus-treated dogs compared with 2/8 (25%) Simparica-Trio-treated dogs, and the GM worm counts were significantly lower (*P* = 0.0316) in Simparica-Trio-treated dogs. Additionally, four (50%) dogs treated with NexGard Plus were positive for *D. immitis* antigen, and one of these four was microfilaremic. In contrast, in the Simparica-Trio-treated group, a single dog was positive for *D. immitis* antigen, and all eight dogs were amicrofilaremic.

**Conclusions:**

Six consecutive administrations of the higher label dosage of moxidectin delivered by Simparica Trio (24–48 µg/kg) protected more dogs from infection with the ML-resistant *D. immitis* isolate ZoeLA than the lower label dosage of moxidectin provided by NexGard Plus (12 – 24 µg/kg).

**Graphical abstract:**

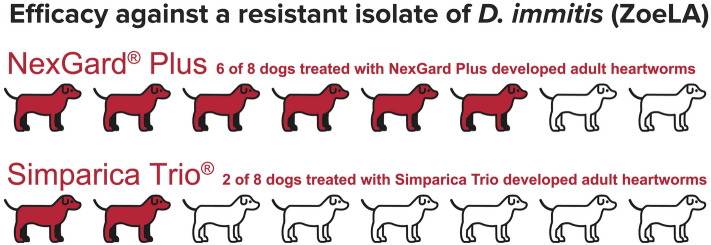

## Background

The filarial parasite *Dirofilaria immitis* is the causative agent of canine heartworm disease, a significant and growing health concern for dogs around the globe [1, 2]. Although therapeutic options have improved over the years, treatment of heartworm-positive dogs remains complex and costly, and it still carries risks of serious side effects [1, 3]. Consequently, the gold standard of care in canine heartworm disease is prevention of infection through the administration of effective prophylactics [1]. All currently available heartworm preventives registered for use in dogs fall into the macrocyclic lactone (ML) drug class, thus the increased evidence of ML resistance creates concern for the future of canine heartworm disease control [4–7]. The degree of resistance appears to vary across ML compounds, formulations, dosages, dose frequency, and resistant isolates [8–16].

The efficacy of MLs is known to be strongly linked to concentration and length of time the drug is present in host tissues [8]. Consequently, studies aimed at controlling ML-resistant *D. immitis* using moxidectin have focused on identifying the optimal dosage and treatment regimen to capitalize on its high potency and long half-life [12, 13]. Simparica Trio^®^ (Zoetis) is a commercially available monthly oral preventive formulated to deliver a minimum dosage of 24 µg/kg moxidectin, 1.2 mg/kg sarolaner, and 5 mg/kg pyrantel pamoate in one chewable tablet. The decision to include moxidectin as an active ingredient in Simparica Trio was based on its higher efficacy compared with other MLs when evaluated against ML-resistant *D. immitis* isolates [8, 10, 13]. It was previously demonstrated that three consecutive monthly dosages of moxidectin at 24 µg/kg provided significantly higher efficacy against ML-resistant *D. immitis* isolates (JYD-34, ZoeMO, and ZoeLA) than did lower moxidectin dosages, as well as comparable efficacy to that provided by three consecutive monthly treatments of moxidectin at 30 µg/kg or 60 µg/kg [12]. The efficacy of moxidectin can be further improved by adding consecutive monthly doses to the treatment regimen, with six monthly treatments at 24 µg/kg providing significantly improved efficacy against JYD-34 than four monthly treatments [13].

The current study further explores the impact of the treatment dosage in the efficacy of moxidectin against ML-resistant *D. immitis* by comparing the preventive efficacy of six consecutive monthly treatments of moxidectin at two different dosages against the ML-resistant isolate ZoeLA in dogs. The study design includes a negative control group; a group treated with six consecutive monthly doses of NexGard^®^ Plus (Boehringer Ingelheim Animal Health), a chewable tablet administered to provide dogs with a minimum dosage of 12 µg/kg moxidectin, 2.5 mg/kg afoxolaner, and 5 mg/kg pyrantel pamoate; and a third group treated with six consecutive monthly doses of Simparica Trio.

## Methods

### Animals

The study used 24 purpose-bred male and female (sex ratio 1:1) Beagles and were required to be ≥ 1.8 kg at the time of infection (day −30) and ≥ 6 months of age at first treatment (day 0). Dogs were healthy, not pregnant or lactating, and not intended for breeding during the study. Each animal was examined on day −35 to determine suitability for study inclusion. All dogs were uniquely identified and had received normal vaccination practice and general care, with no vaccines or medications other than those used in the study administered within 35 days of first treatment on day 0. Dogs were negative for adult heartworm antigen and for microfilaria (including *Dirofilaria* spp. and/or *Acanthocheilonema* spp.) based on blood samples collected on day −35. No macrocyclic lactone had been given to dogs within at least 90 days prior to infection, and none of the dogs on the study had ever received ProHeart^®^ 6 or ProHeart^®^ 12 (moxidectin; Zoetis). Animals were excluded from the study if they were fractious, suffering from disease or injury, debilitated, positive for *D. immitis* microfilariae or antigen on day −35, or otherwise unsuitable for inclusion.

Dogs were housed in 48″ × 60″ runs in compliance with the Animal Welfare Act 9 CFR Parts 1–4 [17] and fed a standard commercially available dog diet, with food offered at least once daily unless fasting was required in accordance with treatment. Water was available ad libitum. Appropriate enrichment was provided, including housing in pairs, and visual and auditory stimuli.

### Study design

The study complied with all applicable animal welfare regulations related to the humane care and use of animals and was approved by the IACUC of TRS Labs, Inc. All animal work was conducted in accordance with local, state, and national regulations. Masking was accomplished by separation of functions of study personnel. All persons making observations, conducting post-treatment heartworm counts, or performing general care for the dogs were masked to the experimental treatments.

The study used 24 dogs, with 8 dogs allocated to 1 of 3 treatment groups (Table 1). Each animal received a physical examination on day −35 including, but not limited to, rectal temperature, thoracic auscultation, skin and hair coat assessment, weight, and the assessment of general physical condition. Additional physical examinations were conducted on days 26, 57, 84, 117, 145, and 236. A blood sample was also collected on day −35 to confirm each dog was negative for heartworm. Each dog was inoculated with 50 *D. immitis* ZoeLA L3 on day −30. The allocation of animals to treatments and pens was according to a randomized balanced incomplete block design with a one-way treatment structure. Blocking was based on pre-treatment body weight (day −7), sex, and pen location. Dogs were moved into their allocated pens on or before day 0. Dogs allotted to T01 were treated with a single tablet of a vitamin–mineral supplement (Pet-Tabs^®^; Zoetis); those allotted to T02 were treated with NexGard Plus; and those allotted to T03 were treated with Simparica Trio. All dogs were dosed orally on days 0, 30, 60, 90, 120, and 150, with dosing based on the most recently collected body weight.

**Table 1:** Group demographics and moxidectin dose administered within each group at each treatment

Treatment	Number of animals	Treatment day	Body weight (kg)		Moxidectin dose administered (µg/kg)		
			Mean	SD	Mean	SD	Range
Negative control^1^ (T01)	8	0	10.5	1.0	–	–	–
		30	10.2	1.0	–	–	–
		60	10.2	1.1	–	–	–
		90	10.3	1.0	–	–	–
		120	10.4	1.0	–	–	–
		150	10.3	0.9	–	–	–
NexGard® Plus^2^ (T02)	8	0	10.0	1.0	17.2	1.6	14.89–18.86
		30	10.2	1.0	17.9	1.7	15.41–19.70
		60	10.1	1.1	17.9	1.9	14.89–20.00
		90	10.4	1.1	17.7	1.8	15.00–19.41
		120	10.5	0.9	17.7	1.7	14.78–19.04
		150	10.5	1.2	17.3	1.6	14.94–19.13
		Overall	-		17.6	1.6	14.78–20.00
Simparica Trio®^3^ (T03)	8	0	11.2	1.0	40.1	7.1	24.11–46.73
		30	10.5	0.9	36.4	9.5	24.11–45.91
		60	10.6	0.9	36.3	9.6	24.22–44.75
		90	11.0	1.0	35.9	9.6	24.11–45.52
		120	11.0	0.9	41.1	7.3	24.79–47.78
		150	11.3	0.9	37.3	8.4	24.11–45.52
		Overall	-		37.9	8.4	24.11–47.78

^1^ Pet-Tabs^®^ Vitamin–mineral supplement administered per label

^2^ Administered as per label recommendation to achieve minimum label dose as follows: 2.5 mg/kg afoxolaner, 12 µg/kg moxidectin, and 5 mg/kg pyrantel (as pamoate salt)

^3^ Administered as per label recommendation to achieve minimum label dose as follows: 1.2 mg/kg sarolaner, 24 µg/kg moxidectin, and 5 mg/kg pyrantel (as pamoate salt)

Clinical observations were conducted on each treatment day (days 0, 30, 60, 90, 120, and 150) by a qualified veterinarian immediately prior to treatment and again at 1 h (± 15 min), 3 h (± 30 min), 6 h (± 30 min), and 24 h (± 1 h) post-treatment. General health observations were typically conducted twice daily but were only conducted once daily on the days physical examinations were performed and on treatment days when multiple clinical observations were performed.

Upon study completion (day 237), all dogs were humanely euthanized using an intravenous administration of a lethal dose of an approved pentobarbital euthanasia solution combined with 1–2 ml heparin (1000 USP units/ml). Animals were allocated randomly to order of necropsy, and any worms present were recorded and recovered. Sedation was administered prior to euthanasia if required. All euthanasia procedures in this study were carried out in accordance with the AVMA Guidelines for the Euthanasia of Animals, 2020 edition [18].

### Infection and treatment

The *D. immitis* isolate used in this study (ZoeLA) is a ML-resistant isolate originally collected from a naturally occurring field case of canine heartworm in Slaughter, Louisiana in June 2013 and validated as a successful infective strain on 10 January 2014 through diagnosis of circulating microfilariae, positive heartworm antigen results, and adult heartworm recovery. The challenge used in this study is the sixth passage of the original isolate, which had been pressured with 3 μg/kg moxidectin orally on the fifth passage. Infective third stage *D. immitis* ZoeLA larvae were harvested from *Aedes aegypti* mosquitoes approximately 14–17 days after membrane feeding on heparinized blood containing 50–100 microfilariae/20 μL (2500–5000 microfilariae/mL). On day −30, each dog was injected subcutaneously in the inguinal region with 50 L3 larvae, and the syringe was rinsed several times with media to ensure all larvae were injected.

All dogs were dosed orally on days 0, 30, 60, 90, 120, and 150. Food was withheld overnight (at least 12 h) prior to each treatment administration. Each dog was offered its regular food ration at least 4 h after treatment administration. Dogs in T01 received a Pet-Tabs chewable vitamin–mineral supplement as a negative control. Dogs in T02 were treated with NexGard Plus per label dosage of 2.5–5 mg/kg afoxolaner, 12–24 µg/kg moxidectin, and 5–10 mg/kg pyrantel (as pamoate salt). Dogs in T03 were treated with Simparica Trio per label dosage of 1.2–2.4 mg/kg sarolaner, 24–48 µg/kg moxidectin, and 5–10 mg/kg pyrantel (as pamoate salt). All dogs were administered 5 mL of water by mouth to encourage swallowing. Dogs were observed periodically for several minutes post-dosing to confirm the dose was swallowed and to monitor for potential adverse events (e.g., drooling, choking, gagging, or vomiting). Additional observations were made of dogs and their pens at 2 h post-treatment for evidence of emesis or product expulsion. Personnel handling dogs used protective clothing and changed clothing between each dog.

### Heartworm testing and worm counts

Blood (~2 mL) was collected from each dog on day −35 (pre-infection), day 180, and day 237 for heartworm antigen (serum) testing and microfilariae (whole blood) count. The SNAP^®^ Heartworm RT Test (IDEXX Laboratories) was used for the detection of adult *D. immitis* antigen, and a modified Knott’s technique was used for the detection of blood microfilariae.

After euthanasia, the pleural and peritoneal cavities of each dog were examined for adult *D. immitis* worms. The posterior and anterior venae cavae were clamped, and the heart and lungs were removed. The precava, right atrium, right ventricle, and pulmonary arteries (including those coursing through the lungs) were dissected and examined for worms. All worms from each dog were counted and identified as male or female, and as either dead or alive. Only worms that were abnormal in both motility and appearance were considered dead; all others were considered alive [19].

### Statistical analyses

Data were summarized and analyzed using SAS^®^ software, Version 9.4 (SAS Institute Inc., Cary, NC, USA). The experimental unit for treatment was the individual animal. Prior to statistical analysis, heartworm counts were natural log-transformed (log_e_[x + 1]). The statistical model for log-transformed heartworm counts was a mixed linear model. Least squares means and standard errors were calculated, and 95% confidence intervals were constructed for each treatment. Geometric means (GM; back-transformed means) were calculated from the least squares means and corresponding back-transformed raw data. Treatment differences were assessed at the two-tailed 5% level of significance (*P* ≤ 0.05), and percentage reduction in worm count for each moxidectin treatment group was estimated using the following formula:

GM percent efficacy = 100 × (GM count (negative control) − GM count (treated)) / GM count (negative control).

## Results

### Animals and dosing

The study utilized 24 Beagles (12 male, 12 female), weighing from 8.5 to 12.5 kg and aged 16 –17 months on day −35. Each treatment group contained four male and four female dogs, and the mean weight and age were similar across treatment groups. All dogs in each treatment group were correctly and completely dosed on each of the six treatment days, and the mean moxidectin dosage administered to each group on each treatment day is presented in Table 1. Across all scheduled treatments, dogs in T02 received mean moxidectin dosages of 17.6 µg/kg, ranging from 14.78 µg/kg to 20.00 µg/kg, and dogs in T03 received mean moxidectin dosages of 37.9 µg/kg, ranging from 24.11 µg/kg to 47.78 µg/kg.

### Worm counts and heartworm testing

Individual dog mean moxidectin dosages, worm counts, and heartworm testing results are in Table 2. Results are summarized by treatment group in Table 3. All dogs in T01 (negative control) were positive for the presence of adult heartworms at necropsy on day 237, with adult worm counts ranging from 26 to 45. As expected, dogs in both T02 (*P* < 0.0001) and T03 (*P* < 0.0001) had significantly fewer adult worms than the negative control dogs, with NexGard Plus and Simparica Trio providing 98.1% and 99.5% efficacy, respectively, compared with T01. All dogs treated with moxidectin had either no worms or only a single adult *D. immitis* detected at necropsy. In particular, a single adult worm was found in six of the eight dogs (75%) treated with NexGard Plus, while only two of the eight dogs (25%) treated with Simparica Trio had a single *D. immitis*, which in one case was a stunted female found in the abdominal cavity (Table 2). The GM worm counts for dogs treated with Simparica Trio were significantly lower (*P* = 0.0316) than for dogs treated with NexGard Plus.

**Table 2:** Individual dog mean moxidectin dose, heartworm test results, and adult heartworm counts

Treatment group	Individual ID	Mean moxidectin dosage (μg/kg) over six monthly treatments	Day 180		Day 237								
			Ag	Mf/ml	Ag	Mf/ml	Adult worm count categorized						Total adult worm count
							Live			Dead			
							F	M		F	M		
Negative Control^1^ (T01)	EKE	0	+	465	+	7866	17		17	0		0	34
	EZF	0	+	1156	+	4500	17		18	0		0	35
	GHF	0	+	1192	+	10,800	19		17	0		0	36
	GIF	0	+	1001	+	8415	20		15	0		0	35
	GYE	0	+	0	+	0	22		19	1		0	42
	HHE	0	+	554	+	4080	19		19	0		0	38
	IJE	0	+	705	+	3910	15		11	0		0	26
	LEF	0	+	2300	+	4505	17		28	0		0	45
NexGard^®^ Plus^2^ (T02)	CGE	18.1	–	0	–	0	0		0	0		0	0
	DWE	18.2	–	0	–	0	1		0	0		0	1
	GDE	19.1	+	0	+	0	0		1	0		0	1
	HDF	17.3	-	0	+	14	1		0	0		0	1
	HQE	18.8	–	0	–	0	1		0	0		0	1
	ILF	15.2	–	0	+	0	1		0	0		0	1
	KBF	19.3	+	0	+	0	1		0	0		0	1
	OEF	15.0	–	0	–	0	0		0	0		0	0
Simparica Trio^®3^ (T03)	CPE	45.3	–	0	–	0	1^4^		0	0		0	1^4^
	HFF	41.9	–	0	–	0	0		0	0		0	0
	HSE	32	–	0	–	0	0		0	0		0	0
	JZE	42.4	–	0	–	0	0		0	0		0	0
	KLE	34.9	–	0	–	0	0		0	0		0	0
	KTF	42.5	–	0	+	0	1		0	0		0	1
	QBF	38.9	–	0	–	0	0		0	0		0	0
	YJF	24.9	–	0	–	0	0		0	0		0	0

^1^ Pet-Tabs^®^ Vitamin–mineral supplement

^2^ Administered as per label recommendation to achieve minimum label dose as follows: 2.5 mg/kg afoxolaner, 12 µg/kg moxidectin, and 5 mg/kg pyrantel (as pamoate salt)

^3^ Administered as per label recommendation to achieve minimum label dose as follows: 1.2 mg/kg sarolaner, 24 µg/kg moxidectin, and 5 mg/kg pyrantel (as pamoate salt)

^4^ Stunted female worm recovered from abdominal cavity

*Ag* antigen, *Mf *microfilaria

**Table 3:** Comparative efficacy of six monthly doses of Simparica Trio versus NexGard Plus against a drug-resistant heartworm isolate (ZoeLA)

Treatment^1^	Dogs positive for *Dirofilaria immitis*			Adult worm counts				
	Antigen^2^ (n)	Microfilariae^3^ (n)	Adult worms (n)	Individual animal	Mean		95% confidence limits	Efficacy^5^ (%)
					Arithmetic	Geometric^4^		
Negative control (T01)	8	7	8	26, 34, 35, 35, 36, 38, 42, 45	36.4	36^a^	28.7 to 45.0	–
NexGard Plus (T02)	4	1	6	0, 0, 1, 1, 1, 1, 1, 1	0.8	0.7^b^	0.4 to 1.1	98.1
Simparica Trio (T03)	1	0	2	0, 0, 0, 0, 0, 0, 1,1^6^	0.3	0.2^c^	−0.0 to 0.5	99.5

^1^*n* = 8 per treatment group. Pet-Tabs^®^ Vitamin–mineral supplement (control); NexGard^®^ Plus, 2.5 mg/kg afoxolaner, 12 µg/kg moxidectin, and 5 mg/kg pyrantel (as pamoate salt); Simparica Trio^®^, 1.2 mg/kg sarolaner, 24 µg/kg moxidectin, and 5 mg/kg pyrantel (as pamoate salt)

^2^SNAP Heartworm test (IDEXX)

^3^Modified Knott’s test

^4^Geometric mean counts with different superscripts are significantly different: *P* < 0.0001 for T01 versus T02 or T03. *P* = 0.0316 for T02 versus T03

^5^Percentage reduction compared with T01

^6^Stunted female worm recovered from abdominal cavity

All dogs were negative for *D. immitis* antigen and microfilariae on day −35. Testing on day 180 showed all negative control dogs were positive for heartworm antigen, and seven of the eight dogs were positive for microfilariae (Table 2). At that time, two dogs in the NexGard Plus-treated group (but none in the Simparica Trio-treated group) were positive for heartworm antigen, and no moxidectin-treated dogs were positive for microfilariae. On day 237, all negative control dogs were positive for *D. immitis* antigen, and seven of the eight were positive for microfilariae. Four dogs treated with NexGard Plus were positive for *D. immitis* antigen, and one of these four was positive for microfilariae. In contrast, seven dogs treated with Simparica Trio were negative for *D. immitis* antigen and microfilariae, and the eighth dog was positive for antigen but was amicrofilaremic.

### Observations and abnormal health events

No treatment-related adverse reactions were recorded for dogs on this study. Observations of abnormal health were generally minor and were similar among the three groups. The most recorded events in dogs included diarrhea (T01 or T02, *n* = 3; T03, *n* = 2) and vomiting (T01 or T03, *n* = 1; T02, *n* = 2). Other recorded events included alopecia, abrasion, dermatitis, superficial wound, and lameness. Most abnormal events resolved on their own and without treatment. The most commonly administered concomitant treatments included antibiotic ointment (T01, *n* = 3; T02, *n* = 1; T03, *n* = 1), nonsteroidal antiinflammatory drug (T02, *n* = 1; T03, *n* = 1), and an antiemetic (T03, *n* = 1).

## Discussion

This study investigated the impact of treatment dosage on the preventive efficacy of moxidectin against an ML-resistant isolate (ZoeLA) by comparing six monthly treatments of NexGard Plus and Simparica Trio. The mean moxidectin dosage in the NexGard Plus group was approximately half of the Simparica Trio group (17.6 μg/kg versus 37.9 μg/kg), resulting in significantly lower worm counts in dogs treated with Simparica Trio compared with NexGard Plus against an ML-resistant isolate, when both products were administered for 6 consecutive monthly doses at the recommended label dose.

All dogs tolerated the treatments well, and no treatment-related adverse events were recorded for any dog. At study end, all negative control dogs were adequately infected (Table 2); however, one control dog was amicrofilaremic at both timepoints despite having a robust mixed sex infection, which is most likely attributed to an immune clearance of microfilariae.

The GM adult worm counts for both moxidectin-treated groups were significantly lower than for negative control dogs (*P* < 0.0001), with all moxidectin-treated dogs being either parasite free or having a single adult heartworm. These results align with prior research showing high levels of protection can be achieved against ZoeLA and other ML-resistant *D. immitis* isolates using multiple consecutive administrations of moxidectin at dosages higher than those required for efficacy against ML-susceptible isolates [12, 13, 15]. However, it is important to note that six of the eight NexGard Plus-treated dogs were positive for *D. immitis* at study end, and one of these dogs was microfilaremic by modified Knott’s test. This was unexpected because only one live adult female was recovered from the pulmonary vasculature of this dog. The most likely explanation is that at least one sexually mature male was present earlier, enabling mating, and that the male(s) either died and were undetectable at necropsy or were alive but not detected. In comparison, only two of the eight Simparica Trio-treated dogs were infected with adult *D. immitis*, and none of the dogs were microfilaremic. This lower presence of *D. immitis* in Simparica Trio-treated dogs is reflected in the statistically lower GM worm count recorded for the group compared with that of NexGard Plus-treated dogs (*P* < 0.0316) and in the higher percent efficacy reported for Simparica Trio (99.5%) compared with NexGard Plus (98.1%). These results add to a recent study directly comparing six consecutive monthly Simparica Trio and NexGard Plus preventive efficacy against the ML-resistant *D. immitis* JYD-34 isolate [16]. Although the percent efficacy against JYD-34 infections was not statistically different between Simparica Trio (99.8%) and NexGard Plus (99.5%), two out of the six NexGard Plus-treated dogs were positive for heartworm compared with one Simparica Trio-treated dog.

This study is one of several studies examining efficacy of oral moxidectin against known ML-resistant isolates of heartworm [12, 13, 15, 20]. Differing results between heartworm efficacy studies are due to biological variability, genetic variability of heartworm isolates, and variability of dosages of drug, drug type, and formulation administered to each dog. The isolate genetics may change over time in a dog, as well as through each passage, with or without drug pressuring, which can have an impact on the susceptibility of the isolate [21]. As the mean dosages of moxidectin administered were similar in both studies, and the geometric mean adult worm counts in the control groups were also similar between this study and the Prullage et al. (2024) study, some of the differences may be attributed to the different heartworm isolates used. In the Prullage et al. (2024) study, JYD-34 was used, which was initially isolated from a dog in Illinois in July 2010. The challenge used was from a third passage, without further pressuring for resistance since isolation from the field (personal communication, JM). The ZoeLA isolate used in the current study was originally isolated in June 2013 from Louisiana and underwent pressuring with a 3 μg/kg dosage of moxidectin on the fifth passage.

The USA is currently the only known region globally with documented heartworm drug resistance, with most known ML-resistant isolates originating from the Lower Mississippi River Valley (LMRV) region and others from the eastern USA [7, 20, 22]. Dogs in areas of circulating ML-resistant heartworm, which are being administered heartworm preventives containing actives that are less or not effective against ML-resistant isolates compared with moxidectin (e.g., ivermectin, milbemycin oxime), are more likely to develop microfilaremic infections and become reservoirs of ML-resistant heartworms [4, 8–10, 13–15]. In the absence of a novel heartworm preventive product, the high efficacy of moxidectin against several ML-resistant *D. immitis* isolates remains an important tool in the prevention of canine heartworm disease. With the increasing threat of heartworm drug resistance, it is, however, important for the veterinary practitioner to understand and consider the effectiveness of currently available moxidectin preventives against resistant isolates. The minimum dosage of 24 μg/kg of moxidectin in Simparica Trio was chosen on the basis of early titration studies against ML-resistant *D. immitis* isolates [12]. Heartworm efficacy studies both in the laboratory and the field, including in areas of known high drug resistance, confirmed its robust efficacy [15, 16, 23]. As confirmed in this study, when six monthly doses are administered, dogs treated with higher dosages of moxidectin, such as in Simparica Trio, have a higher likelihood of remaining heartworm free and are less likely to be microfilaremic, compared with dogs being administered lower dosages of moxidectin. It should be noted, however, that the current and other studies conducted under specific laboratory conditions were evaluating one or a few ML-resistant isolates, whereas dogs in the field may be exposed to larvae of varying levels of resistance depending on their geographic location and exposure levels to mosquitoes [4, 8, 10–16].

In addition to using higher, optimized dosages of moxidectin to reduce infection rates with ML-resistant heartworms, a multimodal approach is important to reduce transmission of ML-resistant isolates. These measures include physical and chemical methods to reduce exposure to mosquitoes (fans, keeping indoors, use of repellents, environmental spraying). It should be noted that the use of sarolaner and fluralaner in controlled studies has also demonstrated that mosquitoes exposed to these drugs in the dogs’ blood are likely to die before being able to transmit infective heartworm infective stages, which can be considered an additional tool in the prevention of transmission of resistant heartworms [24–26].

## Conclusions

In this study, dogs treated with six consecutive doses of Simparica Trio had significantly lower worm counts (*P* = 0.0316) against a resistant heartworm isolate (ZoeLA) compared with NexGard Plus-treated dogs. Treatment with Simparica Trio also protected more dogs from infection with this resistant isolate than did NexGard Plus. In light of the expansion of resistant heartworm isolates, veterinary practitioners should consider the active ingredient and dosage when making recommendations for heartworm disease preventive use.

## Data Availability

Data supporting the conclusions of this article are included within the article.

## Abbreviations

AVMA: American Veterinary Medical Association
GM: Geometric mean
IACUC: Institutional Animal Care and Use Committee
L3: Third larval stage
